# Nanoparticles-Modified Chemical Sensor Fabricated on a Flexible Polymer Substrate for Cadmium(II) Detection

**DOI:** 10.3390/polym10070694

**Published:** 2018-06-21

**Authors:** Nan Wang, Elgar Kanhere, Jianmin Miao, Michael S. Triantafyllou

**Affiliations:** 1 Center for Environmental Sensing and Modeling IRG, Singapore-MIT Alliance for Research and Technology Centre, 1 CREATE Way, 138602 Singapore, Singapore; elgar@smart.mit.edu (E.K.); mistetri@mit.edu (M.S.T.); 2 School of Mechanical and Aerospace Engineering, Nanyang Technological University, 50 Nanyang Avenue, 639798 Singapore, Singapore; jmiao@pmail.ntu.edu.sg; 3 Department of Mechanical Engineering, Massachusetts Institute of Technology, 77 Massachusetts Avenue, Cambridge, MA 02139, USA

**Keywords:** flexible chemical sensor, liquid crystal polymer, bismuth nanoparticles, anodic stripping voltammetry, cadmium detection

## Abstract

This paper presents the development of a chemical sensor which was microfabricated on top of liquid crystal polymer (LCP) substrate. As a result of the unique material properties of LCP, the sensor showed favorable flexibility as well as operational reliability. These features demonstrate potential for integration of the sensor into automated sensing vehicles to achieve real-time detection. The sensor consists of a gold working electrode, a silver/silver chloride reference electrode, and a gold counter electrode. The working electrode of the sensor was further modified with bismuth nanoparticles and Nafion. The modified sensor exhibited a significantly enhanced sensing capability toward cadmium metal ion (Cd(II)) in comparison to the unmodified one. The effects of deposition potential and deposition time on the sensing performance of the sensor were extensively investigated through electrochemical experiments. With optimized parameters, the sensor was capable of quantifying Cd(II) in the concentration range of 0.3 to 25 µg/L. The minimum Cd(II) concentration detected by the sensor was 0.06 µg/L under quiescent deposition. The obtained results suggest that the proposed sensor has a great potential to be deployed for in-situ Cd(II) determination.

## 1. Introduction

Growing concerns regarding water contamination have advanced the development of autonomous and portable vehicles to perform routine in-situ water quality surveillance. Automated sensing vehicles surpass the obstacles faced by the conventional water monitoring approach, which includes labor-intensive sample collection, time-consuming sample transportation, centralized laboratory analysis, etc. Heavy metal ions are one of the most detrimental inorganic pollutants that can be found in the majority of contaminated water samples. Cadmium (Cd(II)), one type of hazardous heavy metal ion, poses a great threat to human health since it accumulates in the body for a long period of time. It has been reported that the biological half-life of Cd(II) in humans is 10 to 35 years [1] upon ingestion. Excessive exposure to Cd(II) causes renal dysfunction [2,3,4], bone disease [5,6,7], and lung impairment [8,9,10]. In general, Cd(II) is brought into surface water by the unrestrained discharge of industrial wastewater as well as localized air pollution. Upon taken by plants from the surface water, Cd(II) can reach to human body through the food chain. Therefore, early detection of Cd(II) level in water is critical to locate the pollution source, thereby preventing long-term exposure.

To achieve in-situ monitoring of Cd(II), it is necessary to incorporate chemical sensors into automated sensing vehicles. Conventional chemical sensors manufactured on solid substrates [11,12,13,14], e.g., silicon, glass, and printed circuit board, are not surface conformal. It requires a relatively large and flat space to mount the sensors due to the rigidity of the substrates, which hinders the hull design of a hydrodynamically efficient vehicle. Hence, the development of chemical sensors on flexible and stretchable substrates is of great interest to both academic and industrial researchers. Liquid crystal polymers (LCPs), a type of thermoplastic polymers offering a high degree of structural flexibility and mechanical durability [15], are promising substrate materials. In addition, LCPs are capable of withstanding attacks from most aggressive acids, bases, and halogenated hydrocarbons [16]. Such extraordinary inertness allows chemical sensors constructed on LCPs to be employed in a harsh environment.

The benefits of adopting LCPs as sensor substrates are also manifested in terms of their high compatibility with standard microfabrication techniques, such as photolithography, sputtering, evaporation, dry etching, etc. A sheet of LCPs can be easily cut into any shape with any dimension, making it feasible to attach them to a normal wafer for manufacturing operations. In this paper, a miniaturized chemical sensor, consisting of a gold (Au) working electrode, a silver/silver chloride (Ag/AgCl) reference electrode, and an Au counter electrode, was fabricated on a type of LCP substrate. To improve the detection ability toward Cd(II), the working electrode was subsequently modified by drop-casting 3 µL solution containing 30 mg/L bismuth (Bi) nanoparticles and 1 wt % Nafion. The reason to choose Bi nanoparticles as the modification material was attributable to the superior alloying capability of Bi to form a low-melting-temperature alloy with Cd(II) [17,18]. Such formation of the fused alloy can promote the accumulation of Cd(II) during the electrochemical pre-concentration process. The sensing performance of the modified sensor was comprehensively investigated by means of electrochemical approaches. Experimental results revealed that the nanoparticles-modified chemical sensor was capable of determining trace levels of Cd(II) with favorable sensitivity, accuracy, and repeatability.

## 2. Materials and Methods

### 2.1. Chemicals and Reagents

All chemicals and reagents used throughout the study were of analytical grade. LCP sheet (ULTRALAM 3850, 100 µm thickness) protected by a copper (Cu) cladding was purchased from Rogers Corporation, Chandler, AZ, USA. Bi-nanoparticles powder (99.9%) was supplied by US Research Nanomaterials, Houston, Texas, USA. Nafion (5 wt % solution in a mixture of water and lower alcohols) was obtained from Sigma-Aldrich, Singapore, and further diluted by absolute ethanol. Bi-nanoparticles solution (30 mg/L) was prepared by dissolving an appropriate amount of powder in 1 wt % Nafion and sonicated for 30 min. Standard Cd(II) stock solution (1000 mg/L) was purchased from Merck, Singapore. Ultrapure water (18.2 MΩ·cm) collected from a Milli-Q system (Millipore, Singapore) was used to dilute the stock solution. Acetate buffer (pH 4.6) obtained from Sigma-Aldrich, Singapore was added to all test solutions as supporting electrolyte.

### 2.2. Fabrication of the Chemical Sensor

**Figure 1:**
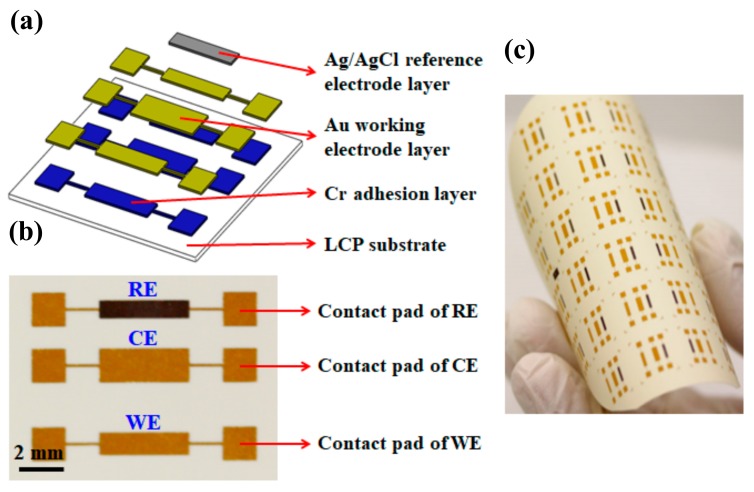
(**a**) Schematic drawing to illustrate the structure of the chemical sensor. (**b**) Photograph of one sensor after fabrication, where ‘RE’, reference electrode; ‘CE’, counter electrode; ‘WE’, working electrode. (**c**) Photograph to show a fabricated sensor array after bending.

As illustrated in Figure 1a, the chemical sensor was fabricated on a piece of LCP substrate. The Cu cladding on the LCP substrate was removed by immersing the entire sheet in etchant for 45 min. Thereafter, the sheet was thoroughly rinsed by deionized water and dried by nitrogen gas. The sheet was then attached to a silicon wafer (4-inch diameter). A layer of 5 µm photoresist (AZ 9260, Merck Performance Materials, Singapore) was spin-coated on the LCP sheet and baked on a hotplate at 110 °C for 4 min. The electrode pattern of the sensor was formed by exposing the photoresist to ultraviolet light (365 nm, i-line) and subsequently by developing in the photoresist developer solution (AZ 400K, Merck Performance Materials, Singapore). After photolithography, a layer of chromium (Cr, 50 nm) was first deposited to serve as an adhesion layer. A layer of Au (300 nm) was then sputtered to function as both working electrode and counter electrode for the sensor. Implementing the same photolithography-sputtering procedure with another photomask, the reference electrode of the sensor was formed by a combined layer of Ag/AgCl (150/250 nm). Figure 1b depicts the photograph of one sensor after fabrication, in which the dimensions of reference, counter, and working electrodes are 4 mm × 0.8 mm, 4 mm × 1.5 mm, and 4 mm × 1 mm, respectively. Each electrode was linked to a pair of contact pads, with which the resistance of the electrode could be measured in order to eliminate defective sensors before further modification and experimentation. The flexibility of the fabricated sensor was demonstrated by completely bending an entire sensor array, as shown in Figure 1c. Even after a few times of bending, no crack/damage was observed on any of the sensors.

### 2.3. Packaging and Modification of the Chemical Sensor

**Figure 2:**
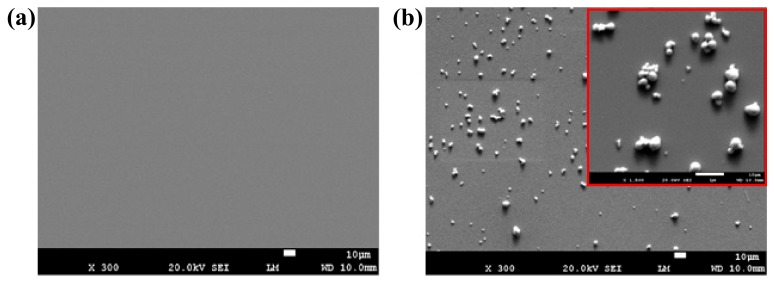
SEM images of the working electrode surface of the chemical sensor (**a**) before and (**b**) after drop-casting Bi-nanoparticles solution, where the inset in (**b**) shows the aggregates formed by Bi nanoparticles with a higher magnification.

To package the chemical sensor, EPO-TEK H20E conductive epoxy (Epoxy Technology, Billerica, Massachusetts, USA) was initially used to connect wires with electrode contact pads (1.5 mm × 1.5 mm) of the sensor. The conductive epoxy was hardened by baking in an oven at 80 °C for 3 h. After cooling, the resistance of each electrode was carefully checked to ensure a stable connection was established. Thereafter, EPO-TEK H70E non-conductive epoxy (Epoxy Technology, Billerica, Massachusetts, USA) was spread on the contact pads to cover both exposed wires and solidified conductive epoxy for insulation. A small amount of non-conductive epoxy was also applied around the working electrode to define a barrier for the succeeding modification process. To harden the non-conductive epoxy, the sensor was baked in an oven at 80 °C for 1.5 h. Finally, 3 µL Bi-nanoparticles solution was drop-casted on the surface of the working electrode and left for evaporation at room temperature for 15 min. Figure 2a,b separately show the scanning electron microscopy (SEM) images of the working electrode surface before and after modification. It can be clearly observed that Bi nanoparticles were evenly distributed on the electrode surface after drop-casting. Some of the nanoparticles stuck together to form bigger aggregates, as depicted in the inset of Figure 2b.

### 2.4. Measurement Procedures

All electrochemical experiments were performed at room temperature using a CHI 600C electrochemical workstation (CH Instruments, Austin, TX, USA). During the experiments, all potentials were measured with respect to the potential of the fabricated Ag/AgCl reference electrode. Cyclic voltammetry (CV) was conducted by immersing the nanoparticles-modified chemical sensor in the solution of 0.1 M acetate buffer. The parameters were chosen as initial potential of −1.5 V, final potential of 0.8 V, and a scan rate of 50 mV/s. Square wave anodic stripping voltammetry (SWASV) was conducted by dipping the nanoparticles-modified chemical sensor in the diluted Cd(II) solutions, which were prepared from the standard stock solution and used without deaeration. The deposition of Cd(II) to the working electrode was achieved under a quiescent condition with deposition potential of −1.0 V (after optimization) and deposition time of 120 s (after optimization). After a 2 s equilibration time, the stripping voltammograms were recorded from −1.2 to 0.6 V with a frequency of 50 Hz, amplitude of 25 mV, and step potential of 5 mV. Prior to next measurement, the sensor was electrochemically cleaned by providing a preconditioning potential of 0.4 V for 2 to 4 min with stirring.

## 3. Results and Discussion

### 3.1. Preliminary Investigation of the Chemical Sensor

**Figure 3:**
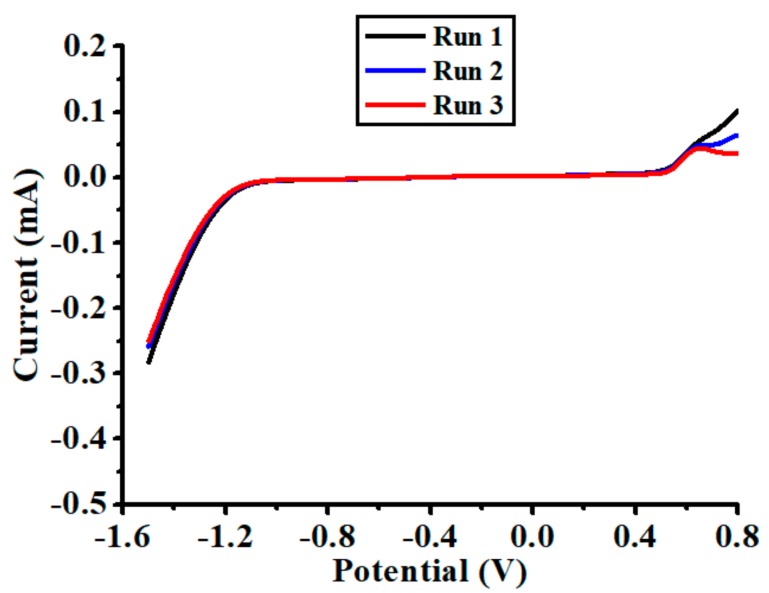
Cyclic voltammograms recorded for the nanoparticles-modified chemical sensor in the solution of 0.1 M acetate buffer, where scan rate is 50 mV/s.

To determine the working range of the nanoparticles-modified chemical sensor, CV experiments were carried out in the solution of 0.1 M acetate buffer. Figure 3 shows the voltammograms recorded in three consecutive runs, in which the current increased dramatically at the negative side when the scanning potential crossed over −1.1 V (toward more negative potential). This implies that a reduction reaction happened in the potential from −1.1 to −1.5 V. Such reaction was possibly attributable to the reduction of hydrogen ions that were present in the buffer solution. On the other hand, the current increased sharply at the positive side when the scanning potential crossed over 0.5 V (toward more positive potential), indicating that an oxidation reaction happened in the potential from 0.5 to 0.8 V. This was possibly due to the partial oxidation of drop-casted Bi back to its ionic state. Therefore, the working range of the nanoparticles-modified chemical sensor was confined between −1.1 and 0.5 V.

**Figure 4:**
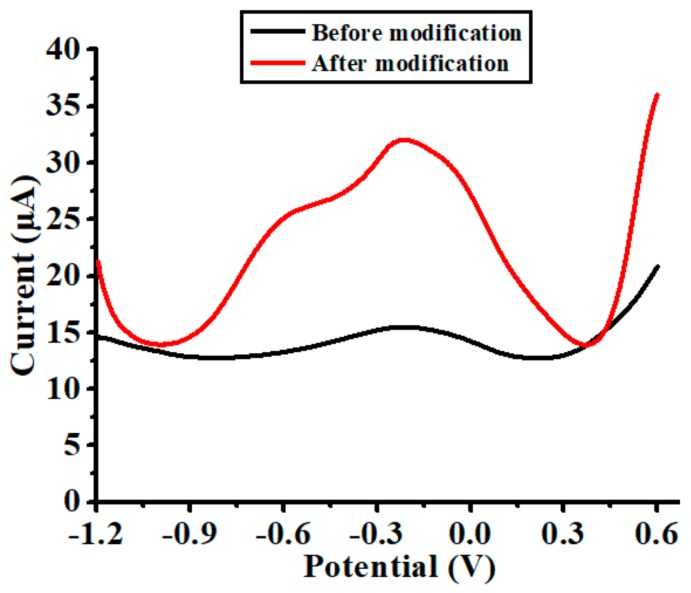
Anodic stripping voltammograms recorded for the chemical sensor before and after modification in the solution of 20 µg/L Cd(II) with 0.1 M acetate buffer, where deposition potential is −0.7 V and deposition time is 60 s.

To evaluate the sensing capability of the nanoparticles-modified chemical sensor, SWASV experiments were conducted in the solution of 20 µg/L Cd(II) with 0.1 M acetate buffer. The outputs of the chemical sensor before and after modification are displayed with black and red lines in Figure 4. It can be clearly observed that the Cd(II) stripping peak was significantly enlarged after modification. In consideration of the magnitude of the peak current (before modification: 2.77 µA; after modification: 18.17 µA), it was augmented by more than six times after modification. This result suggests that the sensing capacity of the chemical sensor after modification was greatly enhanced. Such enhancement was likely attributable to both the increment of surface area (associated with Bi nanoparticles) and the improvement of ion exchange (associated with Nafion). A similar observation has also been reported by other researchers [19,20,21].

### 3.2. Influence of Deposition Potential

**Figure 5:**
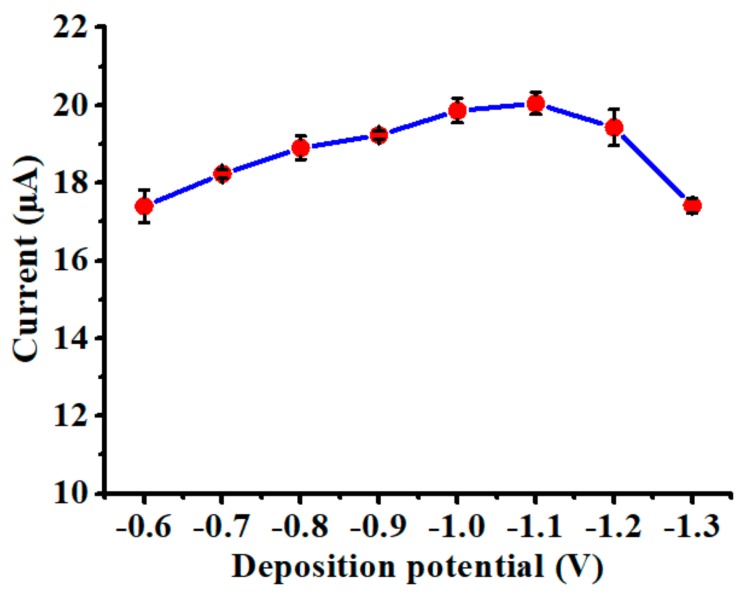
Influence of deposition potential on the stripping peak current of the nanoparticles-modified chemical sensor in the solution of 20 µg/L Cd(II) with 0.1 M acetate buffer, where deposition time is 60 s. Data are presented as the mean of three replicates and error bars denote the standard deviations.

The effect of deposition potential on the sensing performance of the nanoparticles-modified chemical sensor was investigated by performing SWASV experiments in the solution of 20 µg/L Cd(II) with 0.1 M acetate buffer. The deposition time was maintained as a constant value of 60 s for all the experiments. The results are presented in Figure 5, in which the stripping peak current initially increased when the deposition potential was varied between −0.6 and −1.1 V. This was possibly related to the intensification of electric energy provided during the deposition step to accelerate the accumulation of Cd(II). Thereafter, there was a considerable drop of the stripping peak current when the deposition potential became more negative than −1.1 V (between −1.1 and −1.3 V). Such a decrease of peak current was mainly due to the growth of hydrogen bubbles on the working electrode, resulting in a significantly reduced amount of Cd(II) accumulated during the deposition step. The hydrogen evolution at the deposition potential of −1.2 and −1.3 V also matches observations from CV experiments, as shown in Figure 3. To avoid generation of hydrogen bubbles, −1.0 V was selected as the optimal deposition potential for the following experiments.

### 3.3. Influence of Deposition Time

**Figure 6:**
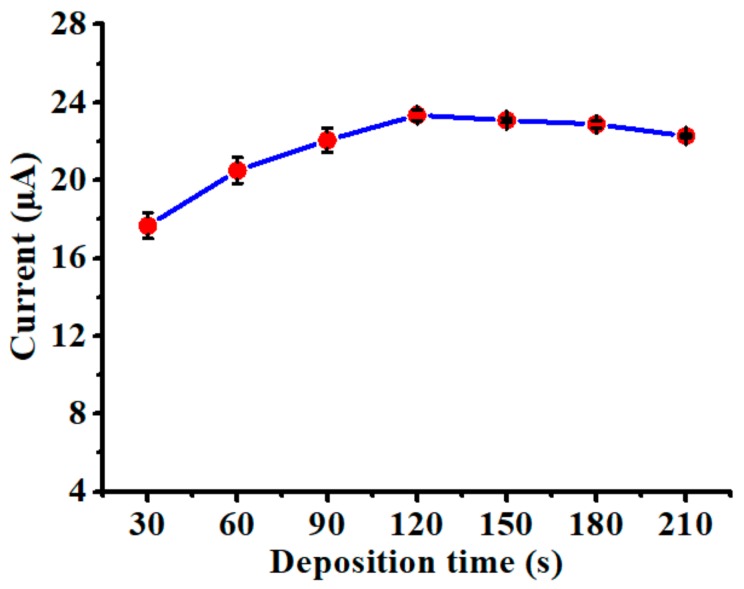
Influence of deposition time on the stripping peak current of the nanoparticles-modified chemical sensor in the solution of 20 µg/L Cd(II) with 0.1 M acetate buffer, where deposition potential is −1.0 V. Data are presented as the mean of three replicates and error bars denote the standard deviations.

The effect of deposition time on the sensing performance of the nanoparticles-modified chemical sensor was explored by performing SWASV experiments in the same test solution (20 µg/L Cd(II) with 0.1 M acetate buffer). As shown in Figure 6, the stripping peak current rose prominently when the deposition time was extended from 30 to 120 s. This was attributable to the increased amount of ions that were able to participate in the deposition process. However, the stripping peak current did not further increase when the deposition time was prolonged from 150 to 180 s. Such stagnation of peak current was likely caused by the saturation of the chemical sensor, considering that the deposition of Cd(II) was conducted in a stationary solution without stirring. The saturation effect may become more severe when the concentration of test solution is elevated. To circumvent possible saturation of the chemical sensor, 120 s was selected as the optimal deposition time.

### 3.4. Analytical Performance of the Chemical Sensor

**Figure 7:**
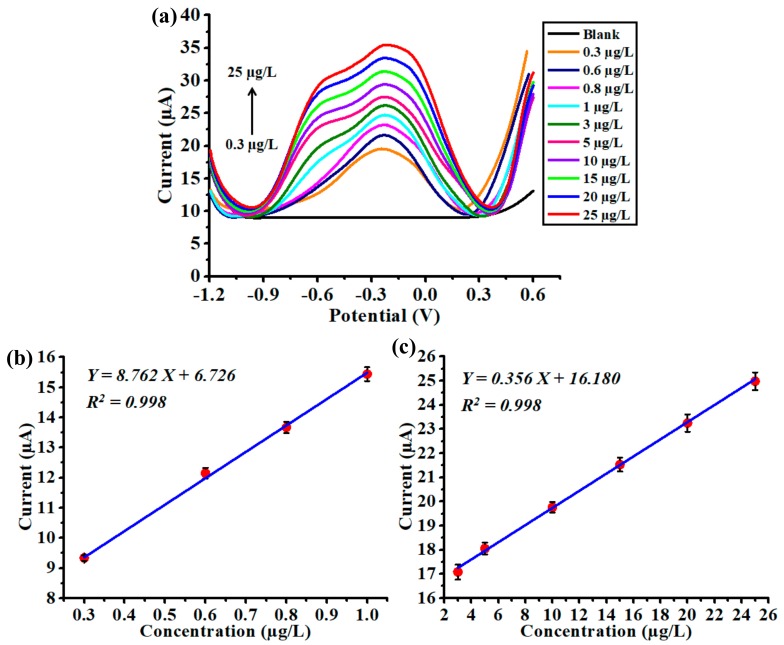
(**a**) Anodic stripping voltammograms recorded for the nanoparticles-modified chemical sensor with increased Cd(II) concentration from 0.3 to 25 µg/L. Calibration plot between the magnitude of stripping peak current and Cd(II) concentration in the range of (**b**) 0.3 to 1 µg/L and (**c**) 3 to 25 µg/L, where deposition potential is −1.0 V and deposition time is 120 s. Data are presented as the mean of three replicates and error bars denote the standard deviations.

Having the optimized parameters of deposition potential (−1.0 V) and deposition time (120 s), the analytical performance of the nanoparticles-modified chemical sensor toward Cd(II) detection was evaluated by conducting a series of SWASV experiments. The Cd(II) concentration in the test solution was increased from 0.3 to 25 µg/L. The sensor’s responses are depicted in Figure 7a, in which all voltammograms were clearly distinguishable and well-defined stripping peaks were easily identified. The minimum Cd(II) concentration, which was able to be detected by the chemical sensor with a legible voltammogram, was 0.06 µg/L. After measuring the magnitude of stripping peak currents, it was noticed that the sensor exhibited linear behavior in two regions, i.e., in a low-concentration region from 0.3 to 1 µg/L, and in a high-concentration region from 3 to 25 µg/L. The corresponding calibration plots are shown in Figure 7b,c, where high values of coefficient of determination (R^2^ = 0.998) were acquired in both regions.

Additionally, the slope value derived from the low-concentration region was much larger than that derived from the high-concentration region. Such variation was probably caused by different accumulation sites for Cd(II) ions during the deposition step. In the low-concentration region, Cd(II) ions tended to be deposited directly onto the surface of Bi nanoparticles because the amount of ions was relatively less in the test solution. However, in the high-concentration region, the amount of ions was significantly enriched, leading to a large proportion of Cd(II) ions to be deposited on the surface of the Au electrode. Considering the alloying capability of Bi toward Cd(II) was much stronger than that of Au [22,23,24], the amount of ions that could be collected by Bi was much more than that of Au.

### 3.5. Evaluation of the Chemical Sensor

**Figure 8:**
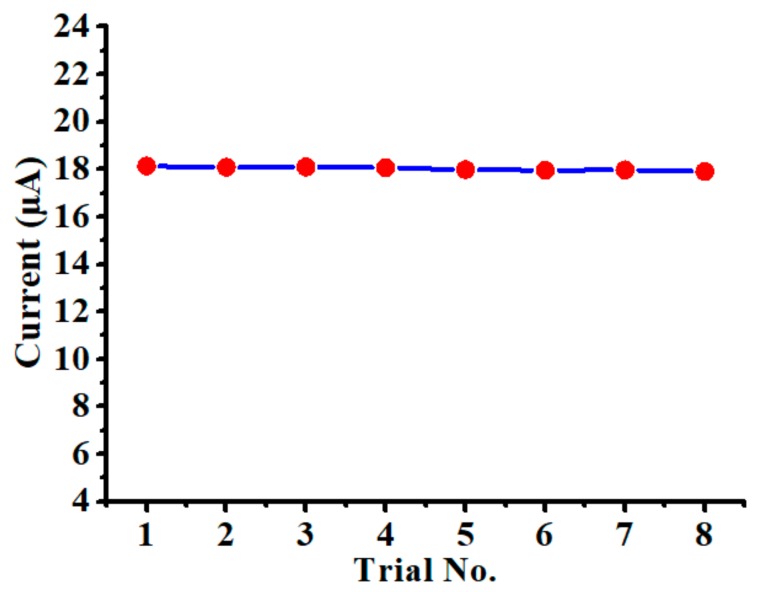
Stripping peak currents measured by the nanoparticles-modified chemical sensor in the solution of 5 µg/L Cd(II) with 0.1 M acetate buffer, where deposition potential is −1.0 V and deposition time is 120 s.

To assess the measurement accuracy of the nanoparticles-modified chemical sensor, eight SWASV trials were consecutively carried out in the solution of 5 µg/L Cd(II) with 0.1 M acetate buffer. The results obtained are plotted in Figure 8, where the sensor displayed consistent responses (18.02 ± 0.081 µA) during eight trials. The relative standard deviation (RSD) calculated among all stripping peak currents was as low as 0.45%, indicating that the sensor was capable of producing repeatable outputs. By employing the calibration equation presented in Figure 7c, the current value can be converted to Cd(II) concentration, which was calculated as 5.169 ± 0.227 µg/L. These results imply that the nanoparticles-modified chemical sensor is applicable for environmental monitoring practice to detect trace levels of Cd(II).

## 4. Conclusions

In this study, an LCP-based chemical sensor modified with Bi nanoparticles and Nafion was proposed, fabricated, and tested for Cd(II) detection. It has been demonstrated that surface modification was exceptionally effective in magnifying the sensing capability of the sensor toward Cd(II) determination. Through electrochemical experiments, the parameters (i.e., deposition potential and deposition time) that affect the accumulation competence of the sensor were optimized. Under the optimal condition, the sensor exhibited favorable analytical performance in terms of a detection range from 0.3 to 25 µg/L, a detection limit of 0.06 µg/L, and repeatability with RSD of 0.45%. The peculiar material properties of LCP endow the sensor to possess both structural flexibility and operational reliability. These merits make the sensor suitable for environmental applications of in-situ Cd(II) monitoring owing to the feasibility of integrating the sensor into automated sensing vehicles.
